# Use of Mueller matrix colposcopy in the characterization of cervical collagen anisotropy

**DOI:** 10.1117/1.JBO.23.12.121605

**Published:** 2018-08-07

**Authors:** Joseph Chue-Sang, Nola Holness, Mariacarla Gonzalez, Joan Greaves, Ilyas Saytashev, Susan Stoff, Amir Gandjbakhche, Viktor V. Chernomordik, Gene Burkett, Jessica C. Ramella-Roman

**Affiliations:** a Florida International University, Department of Biomedical Engineering, Miami, Florida, United States; b Florida International University, Nicole Wertheim College of Nursing and Health Sciences, Miami, Florida, United States; c Jackson Memorial Hospital, Holtz Children’s Hospital, Miami, Florida, United States; d Florida International University, Herbert Wertheim College of Medicine, Miami, Florida, United States; e Eunice Kennedy Shriver National Institute of Child Health and Human Development, Rockville, Maryland, United States; f University of Miami, Leonard Miller School of Medicine, Department of Obstetrics and Gynecology, Miami, Florida, United States

**Keywords:** anisotropy, birefringence, collagen, Mueller matrix, cervix, colposcopy

## Abstract

Annually, about 15 million preterm infants are born in the world. Of these, due to complications resulting from their premature birth, about 1 million would die before the age of five. Since the high incidence of preterm birth (PTB) is partially due to the lack of effective diagnostic modalities, methodologies are needed to determine risk of PTB. We propose a noninvasive tool based on polarized light imaging aimed at measuring the organization of collagen in the cervix. Cervical collagen has been shown to remodel with the approach of parturition. We used a full-field Mueller matrix polarimetric colposcope to assess and compare cervical collagen content and structure in nonpregnant and pregnant women *in vivo*. Local collagen directional azimuth was used and a total of eight cervices were imaged.

## 1 Introduction

With the incidence rate exceeding 11% in the United States and 15%^1^ in the developing countries, preterm birth (PTB), defined as labor prior to 37 weeks of gestation, is the leading cause of infant death worldwide. PTB is reported to be responsible for infant neurological disorders,^2^ long-term cognitive impairment,^3^ as well as chronic health issues involving the auditory, visual, digestive, and respiratory systems.^4^ In expectant mothers, causes for PTB can include infection, inflammation,^5^ vascular disease,^6^ short intervals between pregnancies,^7^ multiple gestations,^6^ and genetic factors.^8^

For an early identification of at-risk pregnancies, as well as to delay the start of labor contractions and thus increase the development time inside the mother, tocolytics, antenatal corticosteroids, and hormones, such as terbutaline, betamethasone, and progesterone, are used. A mechanical approach to delay birth is cerclage, which is used to seal the cervix. Among the current approaches to diagnose PTB are tactile and visual inspection of the cervix to determine dilation and fetal fibronectin immunoassay.^9^ Ultrasound examination is a powerful tool in the prediction of labor and focuses on measurements of cervical thickness.^10^ Shorter cervical thickness is used to identify a higher risk of PTB. However, the lack of positive predictive power of the current PTB diagnosis modalities means that it is difficult for physicians to decide on whether any intervention should be performed. A more reliable diagnosis method could allow physicians to work on delaying birth earlier to give the fetus more development time. This issue also increases the difficulty of developing and testing new treatments. Thus, the development of new diagnostic modalities to identify risk of PTB has great potential in reducing the morbidity of the condition.

Regarding PTB, recent research has highlighted the important role of the cervical collagen of the cervix,^11^^–^^13^ which provides the structure necessary to hold the baby within the uterus during gestation. Numerous studies have investigated the collagen of the cervix to determine how this structure maintains its integrity during pregnancy.^14^^–^^21^ For instance, Aspden et al. found that collagen is oriented in three unique areas surrounding the internal cervical orifice (os), with the anisotropic alignment of the collagen varying within each of the areas. The cervical fibrils are aligned both around and along the cervix os for increased strength.^18^^,^^22^^–^^25^

While the cervix os and outer regions of the cervix are made of collagen aligned in the direction of the cervical canal, the area in-between these regions contains collagen oriented circumferentially around the canal. In this study, this angular measurement of collagen orthogonal to the light path was defined as the collagen azimuth.

**Fig. 1:**
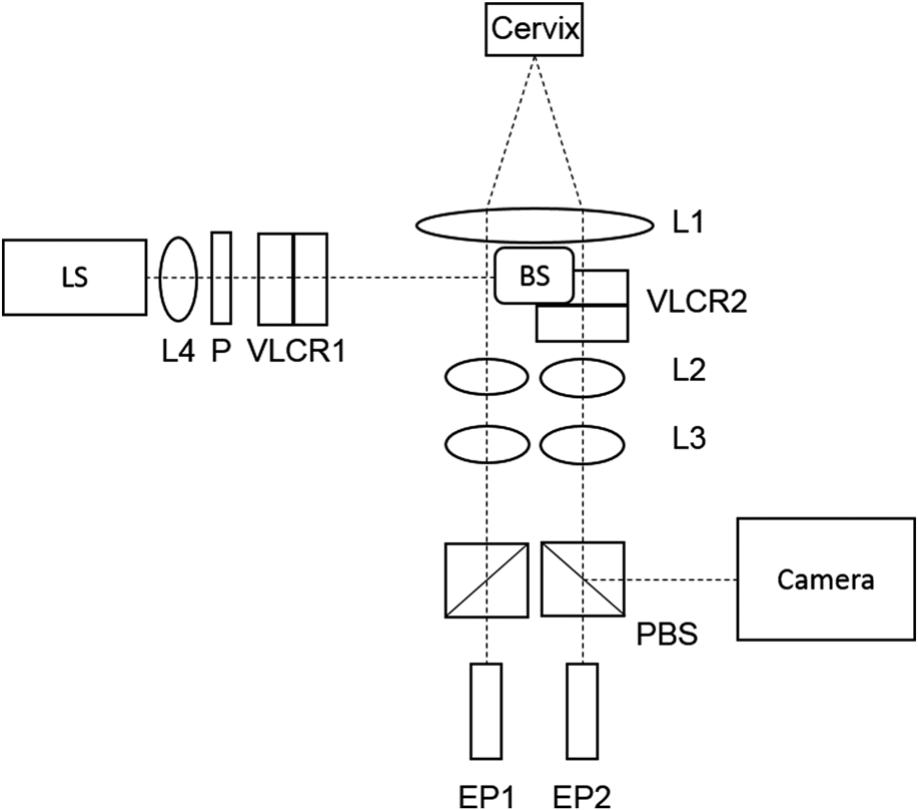
Preterm imaging system. VLCR, variable liquid crystal retarder; P, polarizer; L, lens; PBS, polarizing beam splitter; EP, eye piece; and LS, light source.

Optical measurement of cervical remodeling throughout pregnancy based on the observation of the changes in collagen arrangement and density in the cervix os may help predict changes in the cervix connected to preterm labor. To target the fibrous ultrastructure of the cervix, polarization sensitive techniques, such as Mueller matrix polarimetry, can be used. Relevant research has demonstrated the ability of Mueller matrix polarimetry to identify colorectal and cervical cancer.^26^^–^^28^ We have developed a preterm imaging system (PRIM) based on a standard colposcope, with a high sensitivity to cervical ultrastructure (see Fig. 1). In our previous work, the Mueller matrix methodology was tested on excised porcine cervices, and the results were compared to images produced by optical coherence tomography (OCT).^29^^,^^30^

This work focuses on optical images of the cervix and introduces a modality rather than a full clinical study.

The use of Mueller matrix in the determination of cervical collagen changes has multiple advantages. First, although Mueller matrix polarimetry has a shallow penetration depth, it is capable of measuring collagen in the stroma as we have demonstrated in our previous work.^29^ Studies utilizing MRI of the cervix have shown that the circumferential alignment of collagen present at the vaginal end of the cervix is preserved going toward the uterus end of the structure using MRI.^31^ Second, the PRIM is able to characterize the collagen in the whole cervix with a field of view of up to 3.5 cm, and due to its extended depth of field, it is relatively insensitive to the cervix curvature.

## 2 Materials and Methods

The PRIM is built on a standard colposcope (Seiler Instruments, St. Louis, Missouri) with the addition of polarization optics. Images are acquired with a sCMOS camera (pco.edge, pco., Kelheim, Germany), with lenses L1 to L3 being standard to the colposcope for directing the reflected light from the sample on to the eyepieces and the camera port. A 565-nm LED (M565L3, Thorlabs, Newton, New Jersey) light source is mounted vertically in the illumination port, replacing the fiber optic cable connected to the original white-light source. A linear polarizer (Thorlabs, Newton, New Jersey) and two variable liquid crystal retarders (VLCR1) are mounted after the light source to form the polarization state generator (PSG). Two liquid crystal retarders (VLCR2) (Meadowlark Optics, Frederick, Colorado) and a polarizing beam splitter (PBS) between the camera and L1 form the polarization state analyzer (PSA). In essence, this is the reverse configuration of the PSG. Other groups have used different approaches to polarimetry, such as Vitkin’s use of four photoelastic modulators.^32^^–^^35^

In this study, a total of 16 images were acquired to create a full Mueller matrix following the dual LCVR [variable liquid crystal retarder (VLCR)] approach.^26^^,^^29^^,^^36^^,^^37^ To this end, the VLCRs of the PSG were activated sequentially at four different voltage levels to create four different input polarization states—namely, 0 deg, 45 deg, 90 deg, and elliptical polarization. For each PSG value, a set of four images was acquired by activating the PSA VLCR set at different voltage levels. Calibration of the Mueller matrix polarimetric (MMP) system was performed by a standard methodology previously used by our group^38^^,^^39^ and resulted in the condition number of 3.32. The condition number is defined as the ratio of the largest to smallest singular value decomposition of a matrix. The smaller the condition number the more stable the system as there is less loss of precision. The Mueller matrix of air was constructed with the error below 1%.

### 2.1 Mueller Matrix Decomposition

Mueller matrix decomposition extracts constituent polarization properties from a Mueller matrix of any unknown complex system.^27^^,^^40^ As proposed by Lu–Chipman,^41^ the decomposition of the Mueller matrix (M) yields three canonical matrices accounting for (1) material depolarization ($M_{\Delta }$), (2) retardance due to linear birefringence, and (3) optical activity ($M_{R}$), and diattenuation ($M_{D}$) (see below equation)

$$M=M_{\Delta }M_{R}M_{D}.$$ (1)

Following Sun et al.,^42^ we identified two such parameters relevant to this study: (1) abundance of birefringent collagen δ (retardance) and (2) slow axis orientation θ, related to the orientation of collagen bundles in the tissue. Information decomposed from a Mueller matrix was calculated for each pixel; therefore, we generated parameters of interest of an area after using a median filter on the image. Our MMP resolution was 12.5  μm/pixel and the field of view was up to 3.5 cm. Acquisition time when utilizing image binning could be as low as 1 s for a full set of 16 images, and at full resolution, imaging could take a maximum of 5.6 s.

### 2.2 Image Processing

**Fig. 2:**
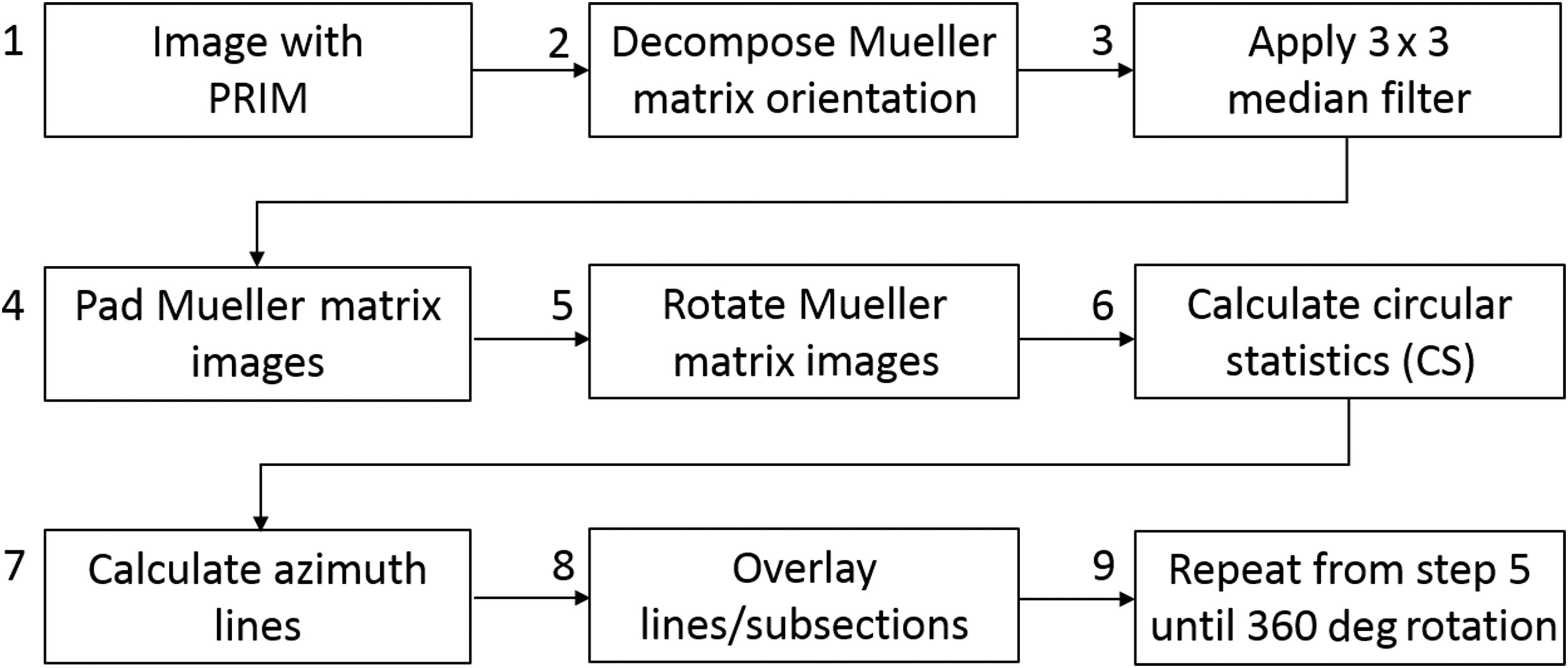
The MMP image processing pipeline.

To improve the quality of the orientation images, nine postprocessing steps were performed (see Fig. 2). First, the movement artifact in an *in-vivo* experiment was considered using the PRIM (step 1). During the acquisition time, large movements were not commonly observed, as the patient was still sitting down with her feet in stirrups from the prior gynecological exam. When a movement occurred, it was mostly in the lateral directions, as it would be caused by the patient’s waist adjusting on the seat. In these cases, ImageJ (National Institutes of Health, Bethesda, Maryland) was used to coregister the image stack without changing the intensity. Due to the low movement artifact incidence, full image resolution could be used. After imaging with the PRIM, the orientation and depolarization data were calculated from the Mueller matrix constructed from the raw intensity images (step 2). A 3×3 median filter was then applied to create a gradual transition of angles around the cervix by mitigating the effect of outlier pixels with a shift in the orientation value (step 3). Thereafter, to ensure that the regions farther away from the cervix os were not cut off with the image rotation, the orientation and depolarization images were zero padded with 500 pixels at each boundary in preparation for rotation around the cervix os (step 4). After manually setting the boundaries of the cervix os, subsections around the cervix were automatically selected by a custom algorithm. This algorithm creates a center point for the cervix from the weighted centroid of the os. From this center point, 50×50-pixel subsections were then generated in the vertical and horizontal directions (step 5). Doing so allowed us to automatically generate histograms of the distribution of collagen azimuth and other statistical analyses without bias in selecting the data, as well as facilitated differentiation of where data points were located relative to the landmarks in the cervices, such as the os. During this process, the cervix os and the area surrounding the cervix were excluded from the actual cervix data. The images were rotated 10 deg each iteration until the complete 360-deg rotation was completed. Steps 6 and 7 were performed on the same set of orientation data. Therefore, the entirety of the cervix could be analyzed using different methods with the same subsections (datasets) (Sec. 2.3 has further detail on circular statistics). The orientation images were analyzed at each iteration by first applying a mask over the data, so that pixels corresponding to a depolarization value <0.5 were not considered in the final analysis (step 8). This removed the areas of saturation caused by specular reflectance from the calculations of the orientation lines that were later projected over the images and allowed no lines to be present in the areas of low depolarization. A retardance threshold mask was also applied to remove the areas of dense mucus (a white buildup in the pregnant cervix, see Fig. 4) from the calculations.

Thereafter, following the methodology of Jan et al.,^43^ a third mask was then applied to the orientation data. The parameter calculated was used to create what Jan et al.^43^ referred to as a weighted polarization “energy” mask, for each pixel depending on its response to the changes in incident polarized light. Pixels with a high energy are generally those with strong birefringence (i.e., higher collagen content) due to their strong response to polarized light. Using this parameter as a gradient mask, it was possible to highlight the areas of strong birefringence while shading areas with progressively less response from the incident polarized light. The areas around spots of low depolarization (saturated pixels) were also shaded by the energy mask. After reducing the noise in the orientation data using the depolarization mask, the originally sized 2560  pixels×2160  pixels orientation image was sectioned into 50  pixels×50  pixels areas and then averaged to calculate the mean angle used to generate their representative lines and overlaid over a grayscale image of the sample. The orientation lines were calculated from the 50×50-pixel regions to give a summary graphic of the mean distribution of the collagen azimuth. The color representation of collagen azimuth in the orientation images already showcased the highest resolution possible for the PRIM system on the pixel-by-pixel basis. Finally, the kurtosis image was then calculated from the orientation data.

### 2.3 Circular Statistics

Circular statistics^44^^,^^45^ is a subset of statistics for the data that can be shown on a unit circle where the sign of values is determined by the direction of rotation, such as in the case of vector coordinates. In this kind of data, 10 deg is identical to 190 deg. Normal arithmetic statistics with these two values would yield a mean of 100 deg when they are in fact the same azimuth angle. Therefore, errors in calculation, such as this, can skew the interpretation of the angular data away from the real retardation axis present. The periodicity of such data requires a departure from the normal arithmetic statistics, which would give a faulty representation of the mean of the dataset. In this study, circular statistics was applied to the orientation data decomposed from the Mueller matrix to calculate the directional parameters of the cervical ultrastructure. This method requires that the data are first transformed into unit vectors with two-dimensional (2-D) data [see Eq. (2)], where θ is the retardation orientation calculated per pixel from the Mueller matrix. Equation (3) is the mean resultant vector $\overset{¯}{r}$ of the dataset. The mean angular direction $\overset{¯}{\theta }$ can be calculated using the four-quadrant inverse tangent of $\overset{¯}{r}$

$$r_{i}=(\frac{\cos\;\theta_{i}}{\sin\;\theta_{i}}),$$ (2)

$$\overset{¯}{r}=\frac{1}{N}\underset{i}{\sum }r_{i}.$$ (3)

In this study, we computed kurtosis and mean angle. Circular kurtosis is the measurement of outliers in a distribution, the distribution’s propensity to produce outliers, and is associated with the weight of the tails in a dataset.^46^ It is useful as a measurement of how unfluctuating a distribution of angles is in a dataset, which can be confounded in mean calculations. A flat distribution of angles where all angles are equal in frequency indicates randomness of orientation and will give a kurtosis of 0, whereas a narrow distribution of angles with few outliers (a small tail) indicates a strongly aligned structure and the kurtosis value will move toward 1. Kurtosis images were generated similarly to how the orientation lines were, but from smaller moving windows of the 5  pixels×5  pixels areas of the orientation images. In this way, a kurtosis image can be generated from the entire cervix with the same dimensions of the depolarization and orientation images calculated from the Mueller matrix. At the same time, kurtosis was also calculated in the 50  pixels×50  pixels regions of the orientation data sectioned in the vertical and horizontal directions centered on the os. An example of these sections can be seen in Fig. 4(a), where the blue sections have a kurtosis above 0.6. This calculation was performed at every 10-deg rotation of the image. The ratio of high-to-low kurtosis sections (KI) was calculated over 360 deg.

## 3 Results and Discussion

To test the ability of our system to ascertain collagen distribution in the live cervix, two different studies were conducted. Study 1 was conducted at the Simulation Teaching and Research Center (STAR Center) at Florida International University (FIU) and focused on healthy nonpregnant women. In this study, the aim was to obtain normative data for further comparison. Approval for *in-vivo* imaging of human patients was granted by FIU’s Internal Review Board (IRB-15-0466-CR01). Study 2 was conducted in the triage unit of Jackson Memorial Hospital, Miami. IRB approval (IRB-16-0244) was obtained both at FIU and Jackson. In study 1, inclusion criteria were nonpregnant woman aged between 18 and 59 years old. Women who were menstruating were excluded because of the difficulty in analyzing the images due to menstruation discharge. Women with abnormal cervices and women who reported themselves to be pregnant were also excluded. In study 2, inclusion criteria were pregnant patients past 24 weeks of gestation and who self-referred themselves to the hospital for the possibility of going into labor. Patients with any kind of vaginal pathology, such as yeast infection, were excluded. The IRB that was approved for this work did not entail a longitudinal study. The pregnant patients were received at an obstetrician–gynecology emergency triage and came in for pain or other risks toward pregnancy. The information we acquired before imaging the patients were their current gestation time, number of previous pregnancies, and for what reason they were admitting themselves to the triage. It is known that the first pregnant patient we imaged went to term due to their admittance at the triage a week prior to labor.

**Fig. 3:**
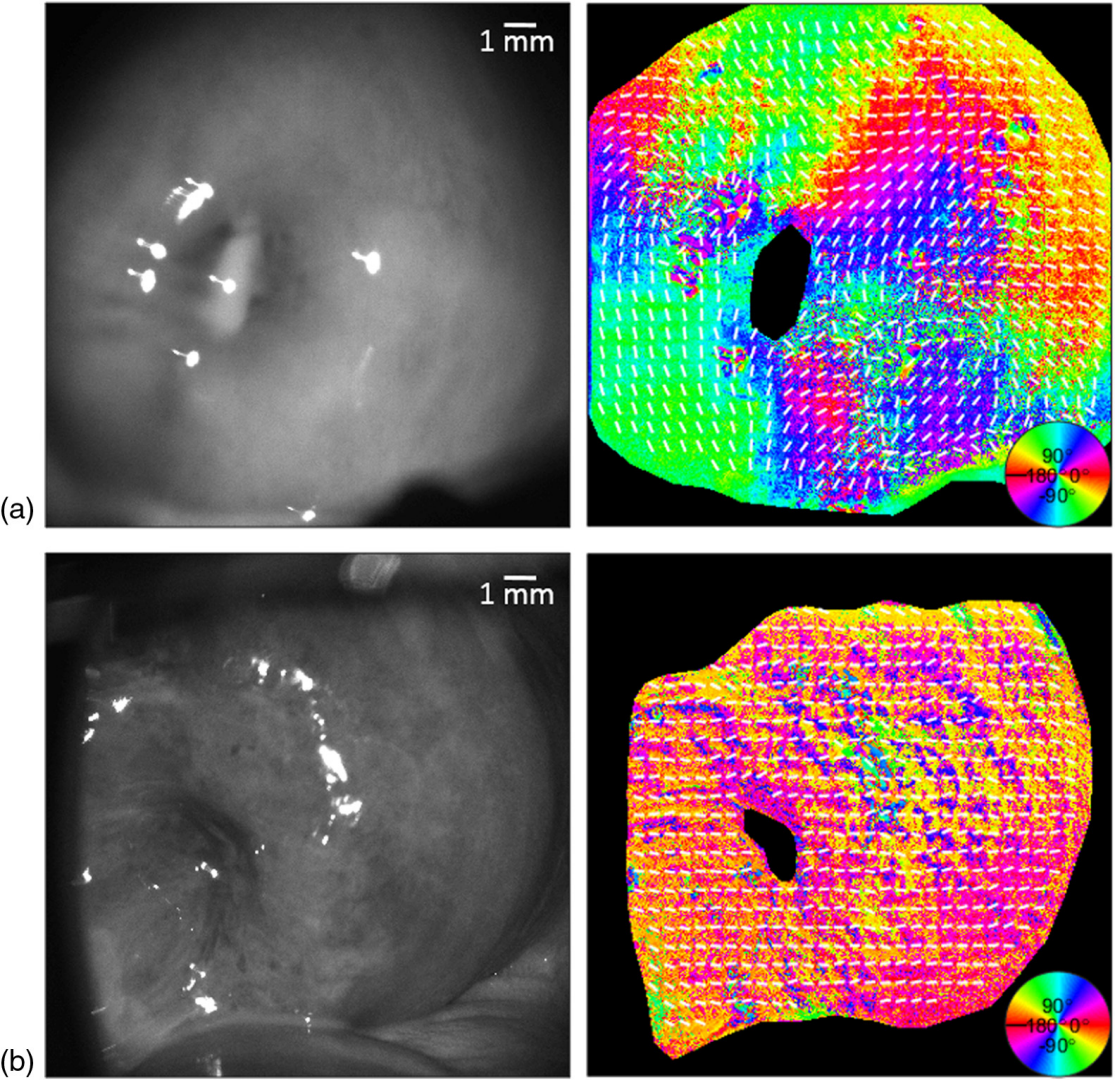
*In-vivo* (a) nonpregnant human and (b) pregnant cervices raw image and MMP decomposed orientation. Circular color bar refers to the retarder orientation calculated from the Mueller matrix of the cervix.

The imaging procedure followed a standard colposcopic examination. The colposcope was positioned at about 10 cm from the patient. The cervix was accessed through a speculum by a nurse, allowing the operator to focus the modified colposcope through the eye pieces. The field of view of the digital images and the eye pieces was coregistered. Up to five sets of images were taken per patient. Each acquisition lasted 5.6 s. If the patient started to feel discomfort for any reason at any time, the examination was canceled and the imaging ended. Representative images are shown in Fig. 3. Orientation of 0 deg is parallel to the horizontal axis and a positive Δθ is considered counterclockwise from horizontal. The circular color bar in the lower right corner of the orientation images corresponds to the change in the retardation axis angle. The white lines overlaid on the image are summary vectors of these angles calculated per pixel.

**Fig. 4:**
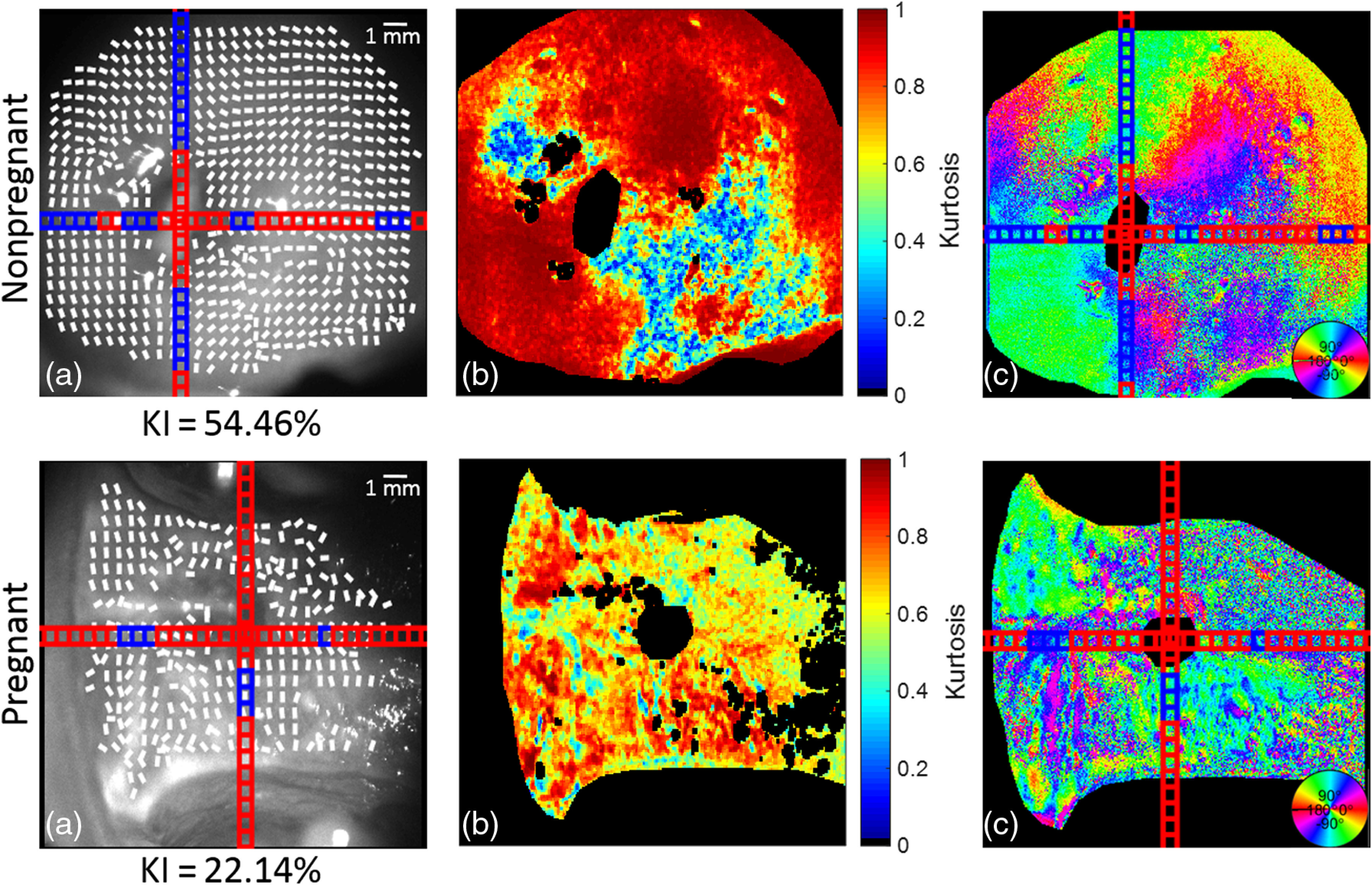
*In-vivo* nonpregnant and pregnant human cervices: (a) B/W CCD image with orientation lines: blue subsections > kurtosis = 0.6 > red subsections, (b) kurtosis, and (c) Mueller matrix decomposed orientation. KI = % of kurtosis values > 0.6 across the entire sample. Circular color bar refers to the retarder orientation calculated from the Mueller matrix of the cervix.

An example of collagen orientation calculated from *in-vivo* images taken from a nonpregnant and pregnant human cervix is shown in Fig. 3. An appropriate shift in orientation angle around the cervical os is designated by the color map. There is a greater resolution of orientation shown by the false color in the nonpregnant cervix compared to the pregnant cervix. This more gradual change in orientation will be shown as higher kurtosis. Mueller matrix decomposition and kurtosis of more samples of human cervices *in vivo* are shown in Figs. 4 and 6.

**Fig. 5:**
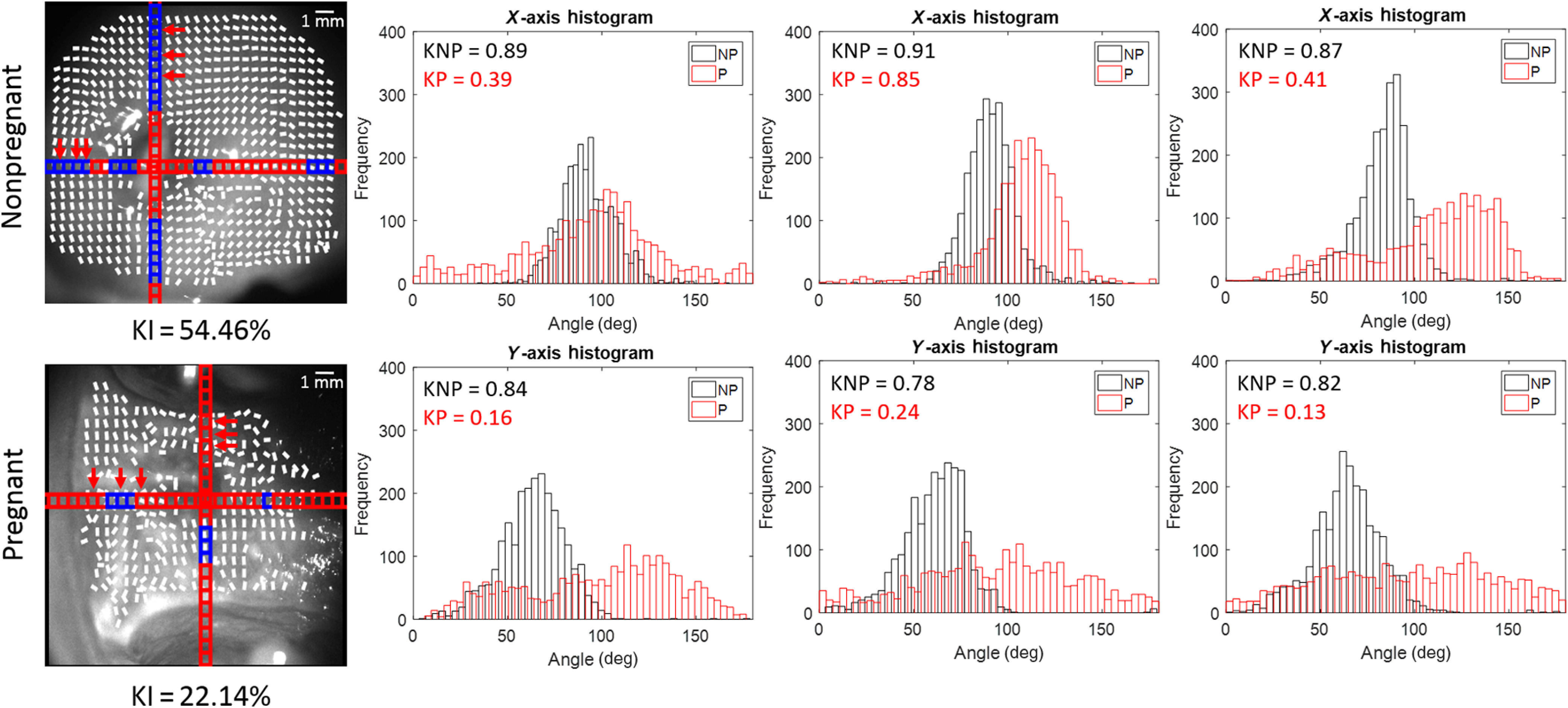
*In-vivo* nonpregnant and pregnant human cervices. The arrows in the grayscale image indicate the subsections on the histogram. Blue subsections > kurtosis = 0.6 > red subsections. X-axis histograms use the subsections going from the left to the right. Y-axis histograms use the subsections going from the top to the bottom. Kurtosis of nonpregnant (KNP) sample; kurtosis of pregnant (KP) sample. KI = % of kurtosis values > 0.6 across the entire sample. There is a poorer collagen alignment in the pregnant cervix as compared to the nonpregnant cervix, as shown by a lower kurtosis and a broader distribution of angles.

A nonpregnant cervix can be seen in the first row of Fig. 4. The areas of low depolarization correspond to specular reflectance and were disregarded from the calculation of orientation lines using the depolarization threshold mask described in Sec. 2.2. This can be seen when comparing the saturated pixels in the grayscale image with the kurtosis image of the sample. There are no lines present over the saturated pixels, which correspond to the blacked-out areas in the kurtosis. The kurtosis values calculated for the nonpregnant cervices mostly range from 0.80 and above as shown by their red color in Fig. 4(b). The areas of lower kurtosis can be found around specular reflectance; however, the kurtosis is generally higher in the nonpregnant sample as compared to the pregnant sample. This is reflected in the 30-percentile difference in the KI value between the two samples. KI was calculated as the ratio between the subsections with kurtosis above 0.6, indicating a strong alignment, and the total number of subsections of the cervix. The single frame shown at 0 deg shows a large discrepancy in the areas with a kurtosis over 0.6, which indicates a better alignment. This can be seen from the color of the subsections. The blue squares indicate the areas where kurtosis exceeds 0.6 as compared to the red squares, which are the areas of a low collagen alignment. A comparison of the orientation data between these nonpregnant and pregnant *in-vivo* human cervices is shown in Fig. 5.

The arrows in the grayscale image indicate the 50  pixels×50  pixels sections, where the histograms were calculated going from the left to the right and from the top to the bottom for the x- and y-axis directions, respectively. While the first row of histograms is in the x-direction, the second row is in the y-direction, as denoted by their titles. To visualize how the orientation distributions compare to each other, nonpregnant and pregnant cervix histograms were plotted together in the same regions from the os. The nonpregnant data are in the black color, and the pregnant data are in the red color. In general, the nonpregnant cervix collagen orientation shown in the black color is more aligned with a tighter distribution of angles and fewer outliers shown by the higher kurtosis and smaller tails in the orientation histogram. This difference in kurtosis is congruent with the expectation, as pregnant cervices should have less collagen fiber alignment over gestation, as well as an increased vascularity, as changes in the cervix occur in preparation to delivery.

**Fig. 6:**
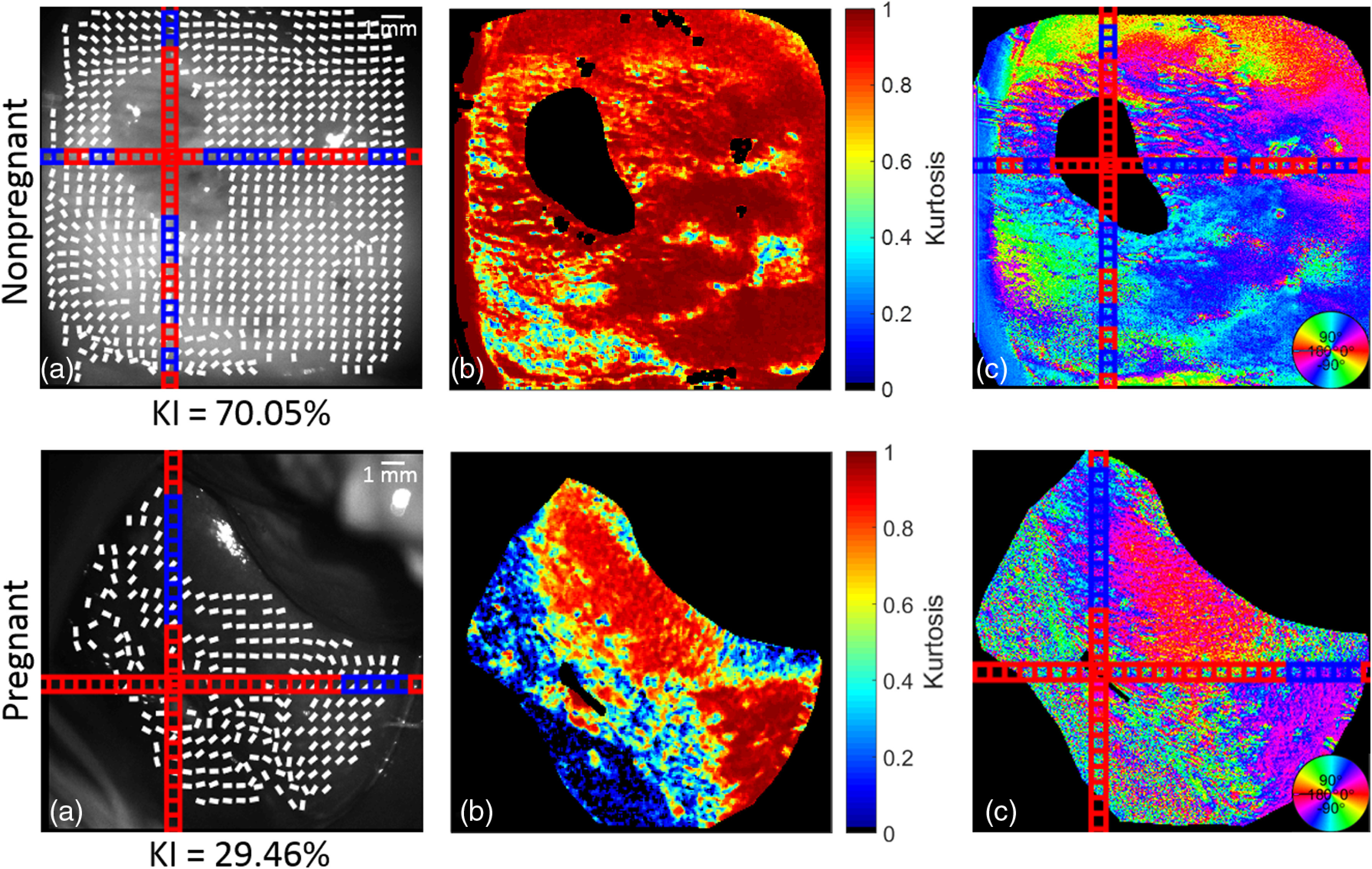
*In-vivo* nonpregnant and pregnant human cervices: (a) B/W CCD image with orientation lines: blue subsections > kurtosis = 0.6 > red subsections, (b) kurtosis, and (c) Mueller matrix decomposed orientation. KI = % of kurtosis values > 0.6 across the entire sample. Circular color bar refers to the retarder orientation calculated from the Mueller matrix of the cervix.

**Fig. 7:**
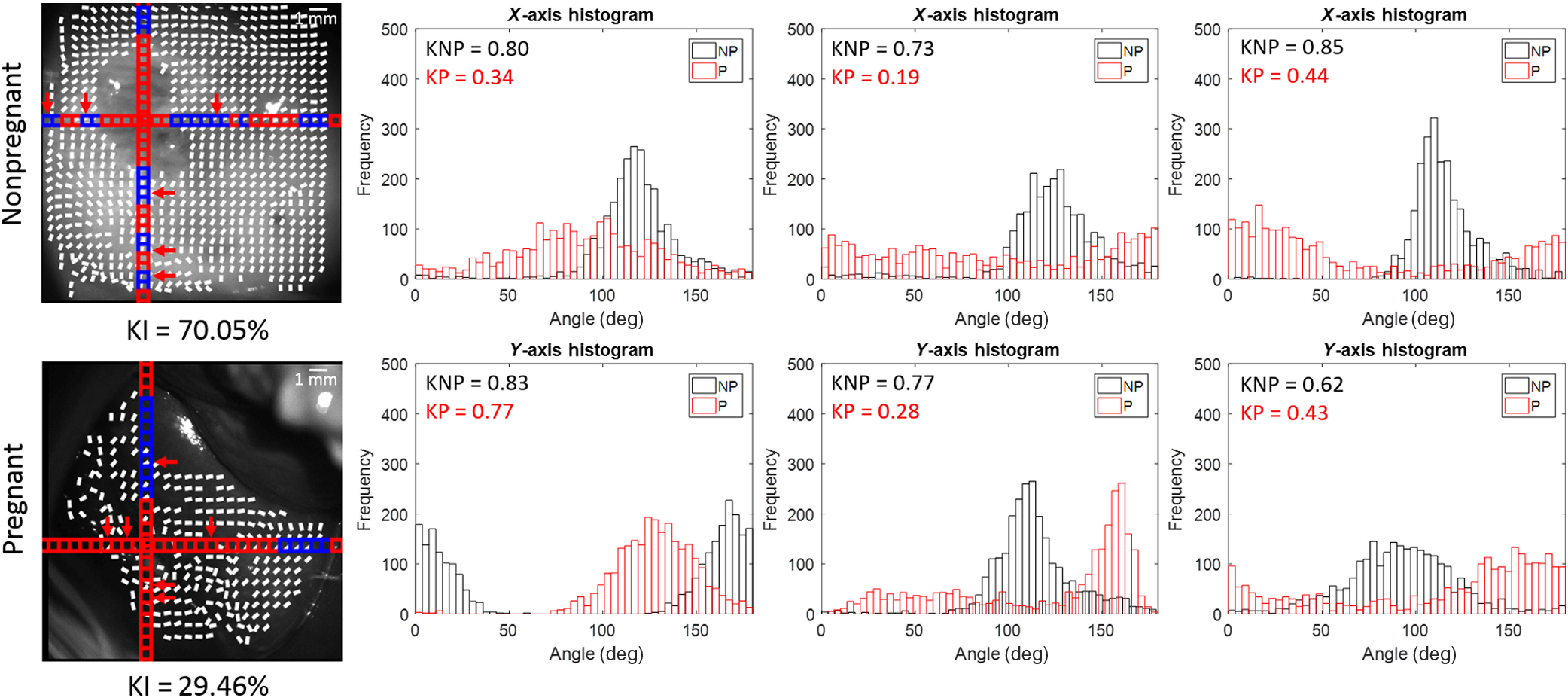
*In-vivo* nonpregnant and pregnant human cervices. The arrows in the grayscale image indicate the subsections on the histogram. Blue subsections > kurtosis = 0.6 > red subsections. X-axis histograms use the subsections going from left to right. Y-axis histograms use the subsections going from top to bottom. KNP sample; KP sample. KI = % of kurtosis values > 0.6 across the entire sample. There is a poorer collagen alignment in the pregnant cervix compared to the nonpregnant cervix, as shown by a lower kurtosis and a broader distribution of angles.

A different set of nonpregnant and pregnant samples can be seen in Fig. 6. Similarly to the set shown in Fig. 5, the areas with specular reflectance were ignored in the calculation of orientation lines, as shown by the gaps in the overlaid lines and the darkened areas in the kurtosis image. Most nonpregnant cervix images show a much higher kurtosis as compared to those of the pregnant sample. This is represented by the 40-percentile difference in kurtosis values above 0.6 between the two samples. Histograms of selected subsections between the nonpregnant and pregnant cervices shown in Fig. 7 provide further evidence in support of the trend of broader distributions of angles in the pregnant cervix that creates a low kurtosis value.

The results of a one-sided T-test on the kurtosis subsections between the nonpregnant and pregnant cervices showed that the mean kurtosis of the nonpregnant subjects was significantly higher than that of the pregnant subjects at the significance level of 95%. After disregarding the subsections that were removed due to the applied masks, the sample size of nonpregnant kurtosis subsections was 918 and 918, whereas that of the pregnant subsections was 882 and 846.

**Fig. 8:**
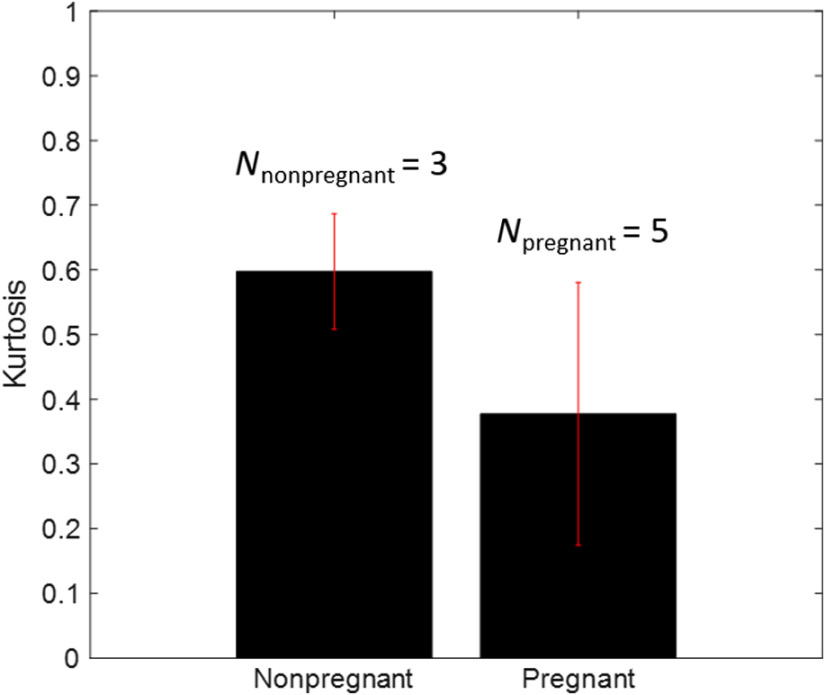
Kurtosis mean and standard deviation of nonpregnant (NP) and pregnant (P) cervices. One sided T-test between both groups showed than mean KNP cervices was significantly higher than that of pregnant cervices, with significance level of 95%.

The mean and standard deviation of the entire cervices are presented in Fig. 8 demonstrating a 20-percentile difference in mean between the two categories. The kurtosis standard deviation is also shown to be greater in the pregnant cervices, likely due to their more randomized arrangement of collagen.

## 4 Conclusion

Optical axis orientation of birefringent materials is essential for the diagnosis of abnormal conditions in tissues with large amounts of extracellular matrix. Depending on the application, tissues that rely on collagen for mechanical strength align the protein in various orientations. Unlike OCT that can yield in-depth image-specific cross sections below the surface of the cervix, the full-field MMP images result from the photon interaction with tissue structures at different depths and are hence an integration of all these interactions. The images presented in this work are 2-D whole field images of the cervix. In this study, we investigated the changes in collagen circumferentially aligned around the cervix os which makes up a large volume of the cervix and can be investigated noninvasively using MMP. In previous research, this circumferential alignment of collagen was found to begin at the surface and continue deeply into the cervix using MRI.^31^

Collagen is important for load bearing in the endocervical canal and can be quickly measured within a set of 16 images needed to create a Mueller matrix. In this study, on introducing an instrument capable of noninvasively imaging the cervix *in vivo*, we determined the collagen orientation within the cervix using Mueller matrix decomposition and several filtering steps. The results of the kurtosis analysis showed an increase in collagen ultrastructure disorganization between nonpregnant and pregnant patient samples. One limiting factor when conducting the measurement was the presence of mucus discharge covering a portion of the cervix. An example of this effect is shown in Fig. 4, where a white film can be seen along the bottom edge of the pregnant cervix. These pixels were excluded, as their retardance and orientation values differed considerably for the areas of the uncovered cervix. The use of different incident wavelengths may reduce this artifact and will be explored in future work; in this study, the cervix was swabbed with a sterile gauze to eliminate the discharge. Further research on collagen orientation in cervices at different time points during remodeling is needed to better understand if Mueller matrix polarimetry can effectively measure changes in cervical collagen orientation in pregnancy or disease.
